# Discourse on medicine: meditative and calculative approaches to ethics from an international perspective

**DOI:** 10.1186/1747-5341-9-18

**Published:** 2014-11-07

**Authors:** David Cruise Malloy, Ronald Martin, Thomas Hadjistavropoulos, Peilai Liu, Elizabeth Fahey McCarthy, Ilhyeok Park, N Shalani, Masaaki Murakami, Suchat Paholpak

**Affiliations:** 1 Faculty of Kinesiology and Health Studies, University of Regina, Regina, Canada; 2 Faculty of Education, University of Regina, Regina, Canada; 3 Department of Psychology, Faculty of Arts, University of Regina, Regina, Canada; 4 Qilu Hospital, Shandong University, Jinan, China; 5 School of Nursing and Midwifery, Trinity College, Dublin, Ireland; 6 Department of Physical Education, Seoul National University Korea, Gwanak District, South Korea; 7 Psychiatric Services and Research Foundation, Chennai, India; 8 Department of Sociology, Meiji Gakuin University, Tokyo, Japan; 9 Department of Psychiatry, Faculty of Medicine, Khon Kaen University, Khon Kaen, Thailand

## Abstract

Heidegger’s two modes of thinking, calculative and meditative, were used as the thematic basis for this qualitative study of physicians from seven countries (Canada, China, India, Ireland, Japan, Korea, & Thailand). Focus groups were conducted in each country with 69 physicians who cared for the elderly. Results suggest that physicians perceived ethical issues primarily through the lens of calculative thinking (76%) with emphasis on economic concerns. Meditative responses represented 24% of the statements and were mostly generated by Canadian physicians whose patients typically were not faced with economic barriers to treatment due to Canada’s universal health care system.

## Introduction

In 1955, the German existential philosopher Martin Heidegger [1] gave his *Memorial Address* in honour of the composer, Conradin Kreutzer (1780–1849). His speech focussed on two kinds of thinking – calculative and meditative. In the former, he argued, we are entrenched; from the latter, we are in flight. These two broadly dichotomous ways of viewing the world are instructive for each of us as they provide frames of reference for engaging others and as well as the physical world. Heidegger cautions us that if we fail to open ourselves to meditative thinking and at the same time allow ourselves to be released from calculative thinking, then we become a “defenceless and perplexed victim at the mercy of the irresistible superior power of technology” (p.52-53). In other words, Heidegger implores us to search for the meaning behind activity – that we can do X does not necessarily imply that we should do X. This scenario is played out daily in medicine as decisions are made to administer or withhold treatment as a function of access to technology and resources.

## Discourse on thinking

Heidegger’s view of ethical thought, as revealed in his *Discourse on Thinking*, suggested that we have the capacity to ponder our decisions in two very different ways. The first he termed calculative, the second meditative. Calculative thinking, he argues,

…computes. It computes ever new, ever more promising and at the same time more economical possibilities. Calculative thinking races from one prospect to the next. Calculative thinking never stops, never collects itself (p. 46).

He likens this mode of thinking with our push toward science, technology, engineering, and mathematical (STEM) solutions to the dilemmas we face personally and professionally, individually and collectively, idiosyncratically and professionally. As an example, he cites a joint proclamation from 18 Nobel laureates who state “Science [and that is modern natural science] is a road to a happier human life” (p.50). Calculative thinking focuses only on utility or immediate functional worth. Starkly, from this perspective, a thing-as-object or a person-as-object has no value unless there is some functionality related to it. He states that “This relation of man to the world as such, in principle a technical one, developed in the seventeenth century first and only in Europe” (p. 50).

This mode of thinking manifests itself in many different forms with regard to our interaction with humans. Perhaps the most obvious is the approach to ethical conduct known as consequentialism. Here in its classical form of act-utilitarianism [2], decisions are made based upon a decision formulae (hedonistic calculus) in which one weighs (in utiles or units of happiness) the relative benefits of action X against action Z. The best choice, the most ‘ethical’ choice is that which results in the measured greatest good for the greatest number. This arguably is the ethical basis for democracy that on the surface is acceptable to most until we trim this view down to its most pernicious possibilities. For example, if we look at the amount of economic loss and physical suffering, to say nothing of heartache that is directly linked with smoking then a rather simple adding of units of happiness could result in the following “modest proposal”:

Government immediately make smoking a capital offence. Over time the incidence of numerous forms of tobacco-related cancer would be eliminated thus saving individuals from a great deal of physical pain and reducing the costs of healthcare and lost time at work by millions annually. The suffering of those initially executed is outweighed by the happiness of the greater number over time that are disease free and economically less burdened by the unnecessary cost of smoking-related healthcare.

This is a logical outcome of the act-utilitarian calculative formulae.

Having said that, Heidegger stresses that calculative thinking and technological advance is needed.

It would be foolish to attack technology blindly. It would be short-sighted to condemn it as the work of the devil. We depend on technical devices; they even challenge us to ever greater advances. But suddenly and unaware we find ourselves so firmly shackled to these technical devices that we fall into bondage with them (pp. 53–54).

Meditative or reflective thinking [3], from which Heidegger argues we are ‘in flight’, is not focussed on utility but rather on meaning. In order to be open to embrace the meaning behind our decisions and actions, we must be able to release ourselves from our calculative, technological, and scientific mind-set. A physician can prolong a life however, are there circumstances where meaningful living or death trumps simply living?

Heidegger believes that our grasp on calculative thinking or rather its hold over us creates a situation in which our meditative or reflective capacities are dulled and meaning hides itself. Many physicians, trained to save lives through science and technology may jump headlong into medical-based solutions with meaning-based solutions available, but hidden from calculative sight.

Releasement toward things and openness to the mystery belong together. They grant us the possibility of dwelling in the world in a totally different way. They promise us a new ground and foundation upon which we can stand and endure in the world of technology without being imperilled by it (p. 55).

In this paper, we explore the relevance of Heidegger’s calculative and meditative modes of thinking in the context of a sample of physicians from seven countries.

## Methods

This qualitative study involved focus groups from seven countries (Canada, China, India, Ireland, Japan, Korea, and Thailand). Countries were selected based upon their standard of healthcare, cultural diversity, concentration of major religions (e.g., Ireland and Catholicism; Thailand and Buddhism) and the availability and willingness of researchers in host countries to participate. Focus groups (n = 15) were held in each country (three in Canada and two in each of the remaining countries) led by the principal researcher with the assistance of translators in non-English speaking countries. In total 65 physicians participated in the study – all of which were recruited via a ‘nomination’ strategy. Research Ethics Boards from each country reviewed and approved the study. Each participant signed a consent form indicating that the researchers would not disclose any information that could identify individuals and that if their statements were used in subsequent publications, they will not be linked to any one participant or focus group. A prescribed list of ten questions was posed to each focus group. This paper focuses exclusively on the first question – *Describe an ethical issue you have faced regarding the treatment of older adults* (note this study is part of a larger international research agenda exploring religion, culture and ethics among physicians). Focus group discussions were audio-taped, transcribed, translated into English and back-translated for verification. Of the 65 participants, approximately 70% identified themselves as medical specialists the remainder were general practitioners.

The lead researcher read the manuscripts and identified 69 statements that were related to physicians’ perspective ethical dilemmas^a^. For example, a Chinese physician stated the following as an ethical dilemma:

I think for myself, if the patient does not have money, and comes to the hospital without money, usually we will send the patients to the leaders of the hospital, and ask permission to do the treatment. This is a very important question for me.

## Results and discussion

Of the 69 statements, 53 (76%) were found to be in the calculative mode and 17 (24%) were in the meditative mode. The ratio was not surprising and reflects what Heidegger might have predicted. What was rather surprising was the dichotomy that was revealed between Canadian physicians and the remaining countries in terms of the relative impact of the financial burden of treatment on patients and their families. Canada generally does not face this problem as a function of the universality of its health care system. In each of the other countries, financial burden was a key and calculative source for ethical dilemmas.

## Calculative thinking

While fifty-two statements could broadly be identified as calculative, the most prevalent theme was *economic burden*. Physicians from all countries within this study, with the exception of Canada, identified the high cost of treatment and the inability of individuals or their families to pay for these services as a dominant ethical problem. The following are examples:

**China**: *Normally most of the patients would give up treatment when they have economic problems*;

**Korea**: *Both patients and guardians (family) want to get service at the minimum expense…older adults indeed want to receive better services but because their financial condition is poor, sometimes they could get into conflict with family*;

**India**: *There is no insurance here in our country to cover for these things. So its really going to pinch their purse…whether to proceed or not becomes a real problem in India, due to the financial reasons*;

**Thailand**: *The ethical concern in the treatment of older patients is different depending on the status of a patient. For example, the patient that is in better status or well educated will be able to pay for medication and receive better treatment. The one that is poor will loose this opportunity of treatment*.

Canadian physicians identified a variety of calculative issues many focussed on the competence of the patient and the strain this creates between physician and patient.

She came in for an injection yesterday (Alzheimer’s patient) and her husband pulled out her SGI (provincial driving insurance) medical form for her driver’s license renewal. I was stunned because I didn’t realise she was still driving.

Canadian physicians identified family tension as an issue (as did other physicians), however the tensions were not rooted in financial concerns, but rather the effort required to broker communication between family members with different views of treatment and withdrawal of treatment.

The situation we find particularly in [Canadian city]…is that there is a far-away-family and the here-family who are in basic front line, and sometimes have different ideas from the far-away-family.

Canadians also identified financial resources as a concern but their focus was not at the individual level of burden, but from the perspective of the healthcare system at large. In the following comment the physician is referring to the family members’ perspective of the continuing treatment of a 98-year-old female suffering from cardiac arrest and pneumonia:

*Should we be using those resources…where there simply are not those resources to use at that point in life. I mean obviously resources have to be moved to a different area* and

*I’ve come across a family whose mother had survived…Nazi incarceration, an assassination attempt, and anyway, a whole lot of things, but the felt that she have suffered so much in her life, that she deserved a little bit of extra time* and *She paid her taxes all her life, so I guess we continue until the end.*

Each of these examples reveals a calculative orientation to physicians’ ethical concerns. They also reflect the decision calculus of ‘happiness’ in stark economic terms (Table 1). Each country has a semblance of government-based health care insurance with the exception of India. Despite this ‘universal’ tone, the coverage for chronic disease (e.g., Alzheimer’s, cancer) varies significantly with each country with Canada providing optimum coverage. The outcome is that before physicians can address broader, arguably more meditative concerns, they must deal initially with whether or not the patient can afford to be treated. Thus the barrier is the financial status of the patient and/or his or her family. Canadian physicians are not required to confront this calculative barrier and thus have the opportunity to ponder ethical concerns that extend beyond the ability and or willingness to pay for needed treatment.

**Table 1:** Background on the health care systems of seven countries

	**Structure of health care system**	**Private vs. Government**	**Waiting lists**	**Medical insurance**
Canada	Universal health care system whereby any Canadian can access essential health services, including medical visits and hospital stays, free of charge.	About 30% of Canadians' health care is paid for through the private sector. There are private clinics that offer some of the same services as the public system.	Universal health care results in waiting lists for certain types of specialists and procedures (although urgent cases are seen quickly).	Many Canadians have private insurance, often through their employer. Private health insurance that could cover procedures in the private sector is not legal.
India	There is government health care free of charge for those in the lowest economic strata. Those from higher strata are charged proportionally to their income.	The central government funds and oversees the universal health policies whereas state governments implement policies at the levels of primary, secondary and tertiary care. There is also a flourishing expensive private medical system.	Waiting lists are rarely encountered, as it is a walk-in system rather than appointments. In the private system, elective procedures are done swiftly (possible due to the competitive market of private health care system).	Medical insurance is not popular – the majority of the population cannot afford it and they have free access to government health care.
	Primary health care facilities are part of a tiered system that funnels more difficult cases into hospitals while providing routine care in the countryside.			
Ireland	The Health Service Executive (HSE) is responsible for the provision of healthcare. Free healthcare is provided for those who earn below a certain income. Currently, free universal GP health care for children under 6 yrs of age is being planned and there is the intention that this will be followed by free universal GP healthcare for persons over 70.	Health care is typically delivered in a mix of private and public systems with most infrastructures being in private hands.	“One per cent of the Irish population is now on a hospital waiting list… The government has already committed £Ir2m this year on its waiting list initiative, but more than 34000 patients are still awaiting reatment…It also pointed out that new statistics show that bed occupancy rates in Irish hospitals were now the highest in the European Union, and, in terms of per capita spending on health among countries in the Organisation for Economic Cooperation and Development, Ireland comes near the bottom, just ahead of Portugal and Greece” [4]	A number of companies offer voluntary private health insurance in Ireland.
				The major provider is the Voluntary Health Insurance Board (VHI) and it is a statutory body whose board is appointed by the Minister for Health. There are also a number of long-established health insurance providers that deal only with particular groups of employees; membership is confined to employees and retired employees and their dependants. These schemes are known as restricted membership schemes.
				The Health Insurance Authority administers a Risk Equalisation Fund which pays health credits to the insurance company for people over 60 to help to meet their higher claims costs. The health credits vary by age, gender and by level of cover. These credits are funded by a community rating health insurance levy paid by health insurers. http://www.citizensinformation.ie/en/health/health_insurance/private_health_insurance.html
Japan	Japan has universal health coverage at a reasonable cost for every citizen. Fees for services are covered by national insurance and public expenditure. The patients pay utmost 30% of the total fee. The financing system is regulated by nationally uniform schedule.	In the Japanese health care system, national and local governments provide healthcare services (including free screening examinations for particular diseases, prenatal care, and infectious disease control).	Reported by the OECD study (2003) to be one of eight countries where the waiting times were low.	People without insurance through employers can participate in a national health insurance program. Since 1973, all elderly persons have been covered by government-sponsored insurance.
Thailand	Thailand has Nation Health Security Policy to ensure that every Thai person can access any essential health services from the local health care center. They are charged a token amount per visit.	Health insurance in Thailand is for profit.	Difficult cases cannot always be treated in local health care centers. Because tertiary health centers are usually located in large provinces or universities, patients might have financial strains when having been referred to these centres.	There are few insurance companies (catering to the needs of expatriates), so competition between companies is hardly present resulting in high prices.
South Korea	Universal health care service is provided under government’s supervision. Any Korean can visit a local hospital or public health clinic for a small of fee for essential medical service.	There are many small private clinics and hospitals as well as public hospitals, but all are supervised by government, and provide similar services. Essential health care costs are same at anywhere.	Waiting lists are very short if any. Doctors in large hospitals are generally overloaded by too many patients.	All insurance is governed and controlled by the government. Private insurance exists only for more excessive health care costs. Insurance fee varies by each individual’s income or wealth. Generally, the employer pays 50% of insurance. However, some high-cost diseases are not fully covered by the insurance. Government pays full healthcare cost for low-income population.
China	There are two main medical insurance policies for non-agricultural population and farm population. The former pay more than latter for their medical insurance, however, the latter receives less coverage for their medical services.	Most of medical institutions are public. Private medical institutions are profit-based.	It is a walk-in system rather than appointment-based in most health care facilities. Appointments may or may not be requested depend on the number of available positions.	Main medical insurance is provided by local governments. There are few commercial insurance.

## Meditative thinking

Of the 69 statements 17 (24%) were deemed to fall into Heidegger’s meditative or reflective theme. These statements demonstrated the physicians’ view that their ethical responsibility extended beyond the mere practice of medicine. In fact, these assertions were philosophical (ontological) in nature as they reflected profound concern with the quality of life and death of their patients. While science was ready-at-hand to respond to physical malfunction, these physicians paused to contemplate the humanity of further treatment.

**Canada:***I think that quite often the quality of life issues are looked at, or certainly viewed, but I think often the quality of dying is disregarded , and I think there is a failure very often in the medical system to try to communicate the value of a positive death experience*; and *We’re really not trained as to exactly, you know, I don’t think its an exact science; its something you have to develop for yourself, to see what works with that and individualise it for the patient himself*; and *Sometimes we get to the point were we feel like we’re torturing our patients, and I think everyone has felt they have tortured their patients…that’s a very difficult situation for a doctor to be in*; and *…in Canada there aren’t endless resources…but I think the whole issue of the rights of the elderly are kind of in conflict with the rights of society*;

**Japan**: In response to the first question that asked to identify a major ethical concern, this Japanese physician responded bluntly: *It is dignity as a human being…I do concern myself with how a human should be treated with respect at the end of her/his life*;

**China**: *Some religion plays an important role. Most Chinese people inherit Confucian ideals - treat old people well and take care for the young. For Muslims, when the patient is going to die…without any operation they just want to take him home. So it’s very easy for doctors to deal with this situation. So religions can help you face this kind of dilemma*;

**Thailand**: *I would like to add…everything relating to them (the patient) must be based on elder dignity*.

These statements reflect a concern that extends beyond the economic and medical feasibility of treatment of the individual-as-host-of-a-pathology (“can we treat”) to concerns about the dignity of the individual-as-human (“should we treat?”). As mentioned early, the economic barrier to medical care in many countries often prevents the physician from travelling down the path of meditative thinking as calculative/economic decisions are made before treatment begins. However those that did reflect about broader non-medical trepidation did so with the added comment that their training was inadequate to address these issues. One Irish physician commented that

I think there is good ethical practice in many ways…my sense of going around the wards being from a fairly homogeneous culture, is that there is a lot of good ethical practice, good ethical care tucked in, but I also think there is often bad articulation of ethical principles. Our ethical is rather underdeveloped and ethical articulacy is low.

## Conclusion

Not unlike other ‘poetic’ philosophers, Martin Heidegger sees our capacity to think as multi-layered and untapped. Aristotle [5] argues that the contemplative life truly distinguishes us from mere animals; Hodgkinson [6] warns us not to rely on the economics and policy of managerialism but to strive for poetic and creative valuation in our role as leaders. Heidegger calls on us to go beyond calculative thought, and engage in meditative thinking. He claims that this is a lost skill that is desperately needed in a world so dominated by technology that humanness and meaning fall victim to pragmatics. The realm of medicine is no stranger to this dichotomy and the findings of this study indicate that healthcare is often driven by the calculative and daunting costs of treatment and medication.

While universal and free healthcare for all is not feasible, advancing medical humanism is worthy of our attention. If Heidegger is correct in stating we are in *flight from* meditative thinking, he is also correct that we are in *flight to* the science, technology, and the economics of medicine. The latter should continue; the former is the responsibility of the medical profession globally to make changes to curricula to give students more than simply an awareness of ethical codes as the basis of ethics education [7]. Students should be encouraged to think about their practice meditatively against the horizon the society and the dignity of the individuals they serve.

### Endnote

^a^English usage in statements by non-English speaking respondents has been corrected by the authors.

## Competing interests

The authors declare that they have no competing interests.

## Authors’ contributions

DM: Data collection and principle author. RM: Analysis of data. TH: Method. PL: Data collection. EF: Data Collection. IP: Data Collection. NS: Data Collection. MM: Data Collection. SP: Data Collection. All authors read and approved the final manuscript.

## References

[B1] Heidegger M Discourse on Thinking 1966 London: Harper Touch Books

[B2] Mill JS Utilitarianism 1861/1957 New York: Bobbs-Merrill

[B3] Krentz A Malloy DC Opening people to possibilities: a Heideggarian approach to leadership Philos Manag 2005 5 1 25 44 10.5840/pom20055129

[B4] Payne D Ireland's waiting list initiative is failing Br Med J 1998 317 1036

[B5] Aristotle Nichomachean Ethics (R.C. Bartlett & S.D. Colins, Trans.) 2011 Chicago: The University of Chicago Press

[B6] Hodgkinson C Toward a Philosophy of Administration 1978 London: Basil Blackwell

[B7] Malloy DC Sevigny P Hadjistavropoulos T Jeyaraj M Fahey McCarthy E Murakami M Paholpak S Lee Y Park I Perceptions of the effectiveness of ethical guidelines: an international study of physicians Med Healthc Philos 2009 12 373 383 10.1007/s11019-009-9212-0 19544088

